# The Association of Hospital Matrons

**Published:** 1919-07-05

**Authors:** 


### THE ASSOCIATION OF HOSPITAL MATRONS.

THE first quarterly meeting of the Association of Hospital Matrons took place on Saturday last at the Medical Society's Rooms, 11 Chandos Street, and a representative gathering of matrons from all parts of England attended, numbering over eighty. The President, Miss Lloyd Still, C.B.E., R.R.C., was in the chair, and amongst those present were Dame Sidney Browne, G.B.E., R.R.C.; Miss M. E. Sparshott, C.B.E., R.R.C.; Miss Gibson, late Matron, Birmingham Infirmary; Miss Girdlestone, R.R.C.; Miss Barton, R.R.C.; Miss Cockrell, R.R.C.; Miss J. E. Hills, R.R.C.; Miss Montgomery, R.R.C.; Miss Cox-Davies, R.R.C. (hon. secretary); and Miss Finch, R.R.C. (hon. treasurer). The meeting was preceded by a service at the Middlesex Hospital.

Miss Lloyd Still, C.B.E., R.R.C., in opening the proceedings, briefly outlined the objects of the Association, which are to maintain the honour and further the interests of the profession, to enable members to take counsel together, to consider and, if necessary, take action upon legislative proposals calculated to affect the interests of the nursing profession, and to justify the existence of the Association by co-operation and the co-ordination of these objects. The reports of the hon. treasurer and of the hon. secretary were read. These showed a bank balance of £42 and a membership of 193. Suggestions of subjects for discussion were invited. The question of the practicability of the report of the Salaries Committee "from the matrons' point of view" was accepted, and placed upon the agenda of the next quarterly meeting.

Miss Macdonald, R.R.C., Matron-in-Chief, C.A.N.S., then gave an interesting portrayal of her impressions of the Nursing Service overseas, and said that "this vital organisation of women, controlled by women, merited the esteem of all, and emphasised the binding relationship of the Canadian Service to the Mother Country." Mr. Leonard Lyle, M.P., who it was hoped would have spoken on Registration, was unavoidably absent. Miss Cox-Davies, in his stead, explained the position of the Nurses' Registration Bill and the proceedings as they had occurred the previous day in the House of Commons. She added that now was the great opportunity for the College of Nursing, and she urged all present individually to exert every effort to further the cause and promote the interests of the College. Her remarks were seconded and supplemented by Miss M. E. Sparshott, C.B.E., R.R.C., and Miss Gibson. The meeting closed with votes of thanks to the promoters of the Association, to the speakers, and a recommendation, which was passed, that a vote of thanks be sent to Mr. Leonard Lyle, M.P., for his kind Parliamentary assistance. Amid expressions of pleasure and goodwill for the future success of the Association the members adjourned for tea.

